# Deep Cytometry: Deep learning with Real-time Inference in Cell Sorting and Flow Cytometry

**DOI:** 10.1038/s41598-019-47193-6

**Published:** 2019-07-31

**Authors:** Yueqin Li, Ata Mahjoubfar, Claire Lifan Chen, Kayvan Reza Niazi, Li Pei, Bahram Jalali

**Affiliations:** 1 Department of Electrical & Computer Engineering, University of California, Los Angeles, California 90095 USA; 2 California NanoSystems Institute, Los Angeles, California 90095 USA; 3 Key Lab of All Optical Network & Advanced Telecommunication Network, Ministry of Education, Institute of Lightwave Technology, Beijing Jiaotong University, Beijing, 100044 China; 4 NantWorks, LLC, Culver City, California 90232 USA; 5 Department of Bioengineering, University of California, Los Angeles, California 90095 USA; 6 Department of Surgery, UCLA Geffen School of Medicine, Los Angeles, USA

**Keywords:** Biomedical engineering, Biomedical engineering, Biophotonics, Biophotonics

## Abstract

Deep learning has achieved spectacular performance in image and speech recognition and synthesis. It outperforms other machine learning algorithms in problems where large amounts of data are available. In the area of measurement technology, instruments based on the photonic time stretch have established record real-time measurement throughput in spectroscopy, optical coherence tomography, and imaging flow cytometry. These extreme-throughput instruments generate approximately 1 Tbit/s of continuous measurement data and have led to the discovery of rare phenomena in nonlinear and complex systems as well as new types of biomedical instruments. Owing to the abundance of data they generate, time-stretch instruments are a natural fit to deep learning classification. Previously we had shown that high-throughput label-free cell classification with high accuracy can be achieved through a combination of time-stretch microscopy, image processing and feature extraction, followed by deep learning for finding cancer cells in the blood. Such a technology holds promise for early detection of primary cancer or metastasis. Here we describe a new deep learning pipeline, which entirely avoids the slow and computationally costly signal processing and feature extraction steps by a convolutional neural network that directly operates on the measured signals. The improvement in computational efficiency enables low-latency inference and makes this pipeline suitable for cell sorting via deep learning. Our neural network takes less than a few milliseconds to classify the cells, fast enough to provide a decision to a cell sorter for real-time separation of individual target cells. We demonstrate the applicability of our new method in the classification of OT-II white blood cells and SW-480 epithelial cancer cells with more than 95% accuracy in a label-free fashion.

## Introduction

Deep learning provides a powerful set of tools for extracting knowledge that is hidden in large-scale data. In image classification and speech recognition, deep learning algorithms have already made big inroads scientifically and commercially, creating new opportunities in medicine and bioinformatics^1^. In medicine, deep learning has been used to identify pulmonary pneumonia using chest X-ray images^2^, heart arrhythmias using electrocardiogram data^3^, and malignant skin lesions at accuracy levels on par with trained dermatologists^4^. The predictive potential of deep neural networks is also revolutionizing related fields like genetics and biochemistry where the sequence specificities of DNA- and RNA-binding proteins have been determined algorithmically from extremely large and complex datasets^5^. Recently, a deep-learning assisted image-activated sorting technology was demonstrated^6^. It used frequency-division-multiplexed microscope to acquire fluorescence image by labeling samples and successfully sorted microalgal cells and blood cells. Moreover, deep learning models helped to analyze water samples so that the ocean microbiome is monitored^7^.

The success of supervised deep learning models, especially convolutional neural networks (ConvNets or CNNs), have fueled research into their application in biomedical imaging^8,9^. By imitating the visual mechanisms of humans and animals to process multiple-arrays data^10^, ConvNets are well-developed in deep learning^11^. The ConvNet models have been successfully applied in the computer vision field such as handwritten digit recognition^12^ and image classification^13–16^. In medical image processing, ConvNets are employed to achieve high-accuracy detection and classification of biological features^17–20^. As another example of the untapped potential of deep learning in accelerating biomedical research, the application of ConvNet models to flow cytometry-derived datasets is introduced in this manuscript.

Flow cytometry is a biomedical diagnostics technique which generates information gathered from the interaction of light (often lasers) with streaming cellular suspensions to classify each cell based on its size, granularity, and fluorescence characteristics through the measurement of forward- and side- scattered signals (elastic scatterings), as well as emission wavelength of fluorescent biomarkers used as marker-specific cellular labels (inelastic scatterings)^21,22^. One application of this technology is fluorescence-activated cell sorting (FACS) which enables the physical collection of cells of interest away from undesired cells within a heterogeneous mixture using multiple fluorescent labels to apply increasingly stringent light scattering and fluorescent emission characteristics to identify and collect target cell populations.

Despite the growing utility of flow cytometry in biomedical research and therapeutics manufacturing, the use of this platform can be limited due to the use of labeling reagents which may alter the behavior of bound cells through their inadvertent activation or inhibition prior to collection or through the targeting of unreliable markers for cell identification. CD326/EpCAM^23^ is one example of the latter. This protein was initially accepted as a generic biomarker for cancer cells of epithelial origin (or their derivatives such as circulating tumor cells) but was later found to be heterogeneously expressed on both or even absent on the most malignant CTC^24^ demonstrating some limitations to this approach. While these findings provide a rationale for the development of label-free cellular analysis and sorting platforms, sole reliance on forward- and side- scattered signals in the absence of fluorescence labeling information has been challenging as a cellular classification modality due to poor sensitivity and selectivity.

As a solution, label-free cell sorting based on additional physical characteristics has gained popularity^25,26^. This approach is compatible with flow cytometry, but entails rapid data analysis and multiplexed feature extraction to improve classification accuracy. To achieve feature expressivity, parallel quantitative phase imaging (TS-QPI) methods are employed^27–30^ to assess additional parameters such as cell protein concentration (correlated with refractive index) and categorize unlabeled cells with increased accuracy.

We have recently introduced a novel imaging flow cytometer that analyzes cells using their biophysical features^31^. Label-free imaging is implemented by quantitative phase imaging^32,33^ and the trade-off between sensitivity and speed is mitigated by using amplified time-stretch dispersive Fourier transform^34–41^. In time-stretch imaging^42,43^, the target cell is illuminated by spatially dispersed broadband pulses, and the spatial features of the target are encoded into the pulse spectrum in a short pulse duration of sub-nanoseconds. Both phase and intensity quantitative images are captured simultaneously, providing abundant features including protein concentration, optical loss, and cellular morphology^44–47^. This procedure was successfully used as a classifier for *OT-II* hybridoma T-lymphocytes and *SW-480* colon cancer epithelial cells in mixed cultures and distinct sub-populations of algal cells with immediate ramifications for biofuel production^31^. However, the signal processing pipeline to form label-free quantitative phase and intensity images and the image processing pipeline to extract morphological and biophysical features from the images have proven costly in time, taking several seconds to extract the features of each cell^48^. This relatively long processing duration prevented the further development of a time-stretch imaging flow cytometer capable of cell sorting because classification decisions need to be made within subseconds, prior to the exit of target cells from the microfluidic channel. Even combined with deep learning methodologies for cell classification following biophysical feature determination, the conversion of waveforms to phase/intensity images and the feature extraction were demanded to generate the input datasets for neural network processing^31^.

To remove the time-consuming steps of image formation and hand-crafted feature extraction, we developed and describe the use of a deep convolutional neural network to directly process the one-dimensional time-series waveforms from the imaging flow cytometer and automatically extract the features using the model itself. By eliminating the requirement of an image processing pipeline prior to the classifier, the running time of cell analysis can be reduced significantly. As a result, cell sorting decisions can be made in less than a few milliseconds, orders of magnitude faster than previous efforts^31^. Furthermore, we find that some features may not be represented in the phase and intensity images extracted from the waveforms, but can be observed by the neural network when the data is provided as the raw time-series waveforms. These hidden features, not available in manually designed image representations, enhance the model to perform cell classification more accurately. The balanced accuracy and F_1_ score of our model reach 95.74% and 95.71%, respectively, for an accelerated classifier of *SW-480* and *OT-II* cells, achieving a new state of the art in accuracy, while enabling cell sorting by time-stretch imaging flow cytometry for the first time. Additionally, our technique for real-time processing of signals by deep learning can be used in other optical sensing and measurement systems^49–55^.

## Results

### Data preparation

**Figure 1:**
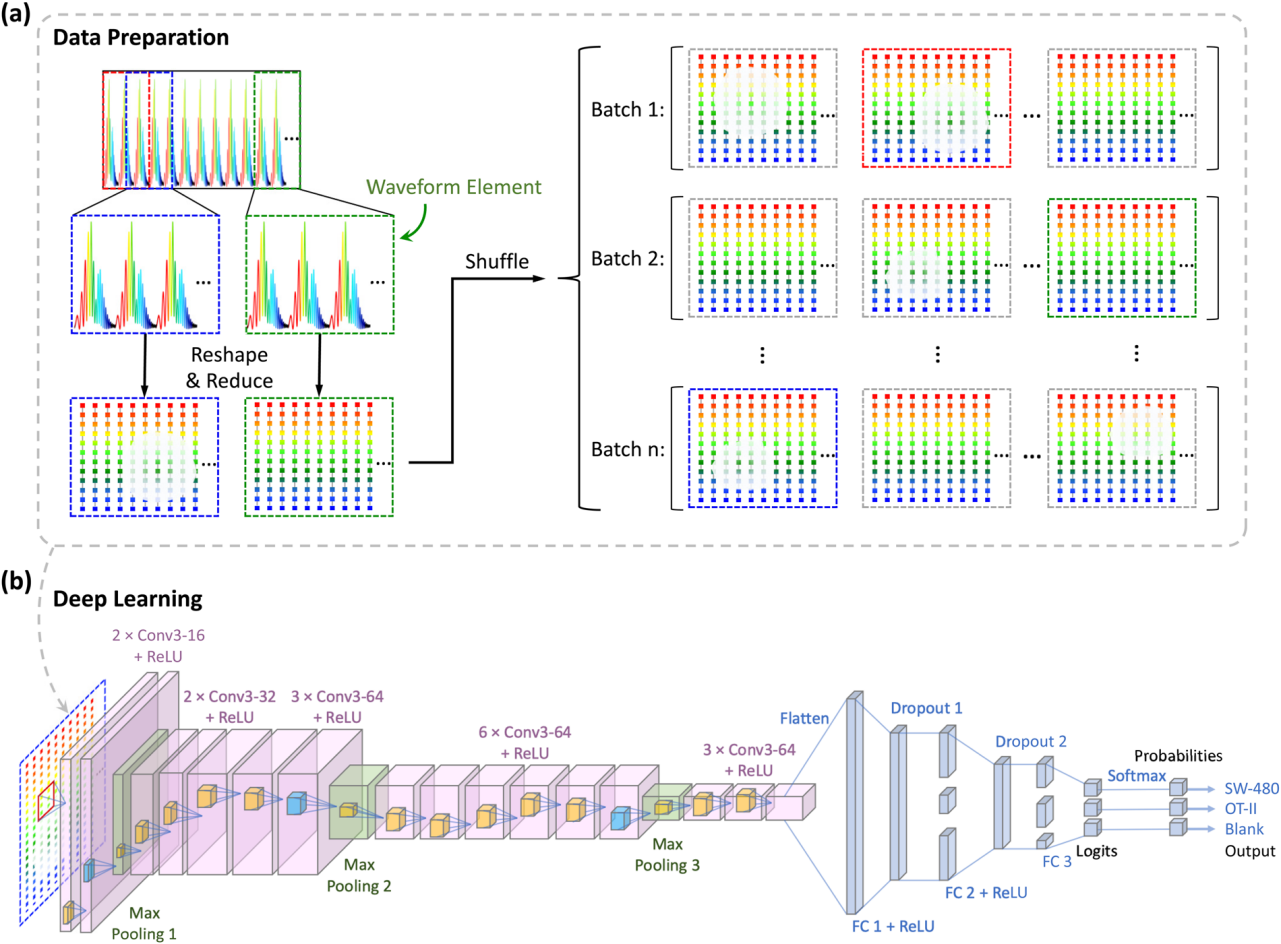
Data preparation and deep learning pipeline. (**a**) The creation of the dataset. The raw TS-QPI waveform files collected by the ultrafast ADC are used as input data directly without conversion to images. Each waveform is divided into 100 waveform elements with an overlap ratio of 50%, creating the redundancy to enhance the training stability. At the beginning, these waveform elements are one-dimensional time-series data. To fit with the conventional convolutional neural network architectures, the waveform elements are reshaped into two dimensions: one dimension corresponds to the laser pulses in each element, the other dimension corresponds to the sampling points per pulse. To shorten the processing time, the digital resolution is further reduced by a reduction factor of 40 in the first dimension of the reshaped waveform elements. The resulting dataset composed of reshaped and reduced waveform elements is fed into the deep learning model as input examples. The whole dataset is split into three subsets consisting of training, validation, and test datasets. Since the entire dataset is too large to be processed at once due to the memory limitations, only a batch of examples is loaded and learned by the model at each iteration. (**b**) Architecture of the learning model. The deep convolutional neural network model (inspired by VGGNet) consists of 16 convolutional layers, three max pooling layers, and three fully-connected layers. The convolutional layers extract and learn the features of the input examples with 3 × 3 kernels (m × Conv3 − p + ReLU stands for m convolutional layers with p output filters and ReLU activation functions). Then max pooling is performed to reduce the number of parameters and computations. The first two fully-connected layers have full connections to all nodes in the previous layer and both apply dropout regularization after them. The third fully-connected layer computes the logits, which are the unscaled log probabilities of the three categories, namely *SW-480* colorectal cancer cells, *OT-II* hybridoma white blood cells, and running buffer alone (blank examples). Finally, the probabilities of the three categories are output after a softmax layer, and the input example is classified.

As a first step towards data preparation, the spatial information of cells is mapped into one dimensional time-series data by time-stretch imaging technology and collected by an analog-to-digital converter (ADC). Without image processing and manual feature extraction, we directly use these raw waveform files as input data for cell classification, decreasing processing time to a scale consistent with decision times used in standard cell sorting. To augment the dataset and perform a sliding window object detection, each time-series waveform is divided into 100 smaller time-series (here referred to as waveform elements) with an overlap ratio of 50% (Fig. 1a). So, the length of each waveform element is 2/101 of the originally acquired waveforms. The input dataset is generated from these waveform elements, and therefore, the number of examples in the input dataset is 100 times larger than the number of waveforms acquired. These elements are further processed to ensure that they initiate from a full pulse (see methods for details of the laser pulses used in time-stretch imaging). The waveform elements are reshaped to two-dimensional arrays, which resemble conventional images, relaxing waveform analysis to an equivalent image classification task for convolutional neural networks.

Since optical resolution measured by the knife-edge method (imaging a target forming a spatial unit step function) is 2.5 *μm*, and the system under study uses a laser with a 36.6 MHz repetition rate and a microfluidic channel with 1.3 m/s cell flow rate, there exists a redundancy, where the number of pulses imaging the target within the resolution distance is greater than one. This redundancy helps to reduce the system’s noise and improves accuracy. However, this redundancy also imposes the use of more memory which concomitantly increases the processing time. To balance the trade-off between accuracy and processing time, a pulse reduction factor of 40 was used to retain every other 40th pulse in a waveform element. In other words, 39 out of every 40 consecutive pulses in a waveform element are removed in the digital domain, similar to discarding 39 columns of pixels for every 40 columns in an image; this reduction in resolution simultaneously decreases the memory footprint of each waveform element and speeds up the computation, while maintaining high-levels of accuracy. The reshaped and reduced waveform elements are the input examples carrying the information of *SW-480* cells, *OT-II* cells and blank areas with no cells. These examples in the dataset are initially shuffled and then randomly divided into three subsets: the training dataset (80%), the validation dataset (10%) and the test dataset (10%), so that there is no overlap between any of these three subsets.

Due to practical memory limitations, only batches of the training dataset can be evaluated by the neural network during every iteration. For this purpose, the batch size is set to 64 examples per training iteration, which results in stochastic optimization of the network parameters. To complete one epoch, batches of the examples are consumed until the entire dataset is processed once by the network. At the end of each training epoch, the performance of the network is evaluated by the validation dataset. Before the next epoch starts, the data in the training, validation, and test datasets are reshuffled independently.

### Model architecture

Since convolutional neural network architectures are good at spatially-correlated feature extraction, we also design a convolutional model inspired by VGGNet^14^ for cell detection and classification. In a convolutional layer, the features are extracted from the input by sliding filters with convolution operations, generating feature maps correspondingly. The model consists of 16 convolutional layers with strides of 1 and kernel sizes of 3 × 3, where the feature depth gradually increases from 16 to 64 output channels (Fig. 1b). In between the convolutional layers, down-sampling is performed by three max pooling layers with a 2 × 2 window size. In these max pooling layers, the dimensionality of the layer is reduced by retention of only the maximum values within the subregions. These values also provide the most critical information. The output from the last convolutional layer is flattened to one dimension. Then three fully-connected layers are attached immediately after: first two have 1024 and 64 nodes, respectively, and dropout regularization is applied to them; the third one produces the unnormalized logits for the three categories to be classified. Finally, the predicted probabilities of the classes are obtained by a softmax layer from the logits. By using these probabilities, the cross-entropy error can be calculated and minimized by the Adam optimizer^56^ during back propagation and the variables of the model are updated iteratively. To introduce nonlinearity, all convolutional and fully-connected hidden layers are equipped with Rectified Linear Unit (ReLU)^10,57^.

### Convergence of the learning process

**Figure 2:**
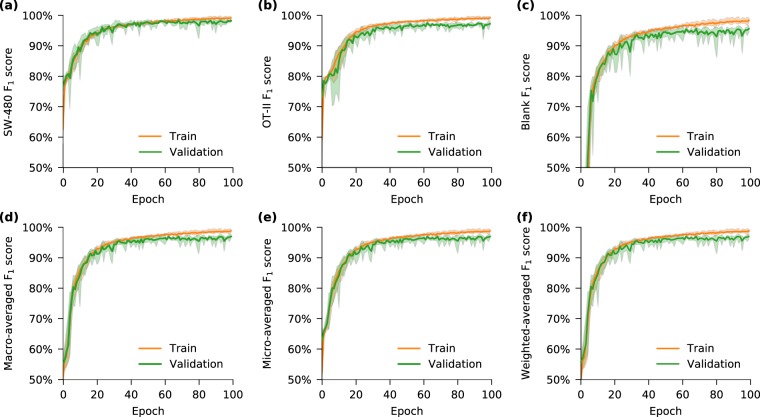
Convergence of the network training. F_1_ score, as a measure of the classification performance, is shown for individual classes (**a**–**c**) and their averaged (combined) forms (**d**–**f**) over training epochs. At each epoch, the network is trained with all examples in the training dataset, and its performance over these training examples is averaged to obtain the training F_1_ score of the epoch (orange curves). At the end of each training epoch, the network is used for classifying all examples in the validation dataset resulting in each epoch’s validation F_1_ score (green curves). This neural network succeeded to recognize (**a**) *SW-480* cells and (**b**) *OT-II* cells even at the end of the first train epoch, but required additional runs to detect (**c**) regions of the waveform containing no cells (blank examples). The shaded area demonstrates the range of performance variations in each epoch for five different training runs. The validation performance approximates the training performance, indicating the model is well-regularized.

In order to better study the learning behavior of the neural network model, the performance of each class and their averaged forms are evaluated for every epoch on the training and validation datasets (Fig. 2). There are multiple ways to measure the performance of the model; tracking the F_1_ score is one such example. The F_1_ score is the harmonic mean of precision and recall, where precision is the positive predictive value measuring the correctness of the classifier and the recall measures the completeness. Therefore, F_1_ score is considered a very effective means of measuring classification performance. In addition to the F_1_ score, the balanced accuracy of the model measured over epochs is also calculated and provided in the Supplementary Information (Supplementary Note 2: Balanced accuracy of the training). Since the examples in the dataset are categorized into three classes (*SW-480*, *OT-II* and blanks), the task for the neural network is multi-class classification as evaluated by calculating the F_1_ score per class and also their averaged forms. Three forms of F_1_ score averaging are taken into account: (1) the micro-averaged F_1_ score, which considers aggregate true positives for precision and recall calculations; (2) the macro-averaged F_1_ score, which evaluates precision and recall of each class individually, and then assigns equal weight to each class; (3) and the weighted-averaged F_1_ score that assigns a different weight to each class should the dataset be imbalanced. Orange curves show the train F_1_ score while green curves show the results of validation F_1_ score. Comparing the classification performance for each class, this neural network demonstrates successful recognition of *SW-480* colorectal cells and *OT-II* hybridoma T cells upon completion of the first training epoch. Interestingly, classification of the acellular dataset require approximately 10 epochs to achieve similar performance. The overall performance is determined by the averaged F_1_ scores of these three classes. The F_1_ scores of the training and validation datasets continue to improve until a maximum is reached at approximately the epoch 60. Meanwhile, the close performance of the train and the validation sets reveals a good generalization of the model. Ultimately, the weighted-averaged validation F_1_ score achieved 97.01%. To evaluate the reproducibility of the results obtained by this neural network, the training procedure was repeated five times starting from randomly initialized weights and biases and demonstrated significant concordance between runs. The standard deviation of the weighted-averaged validation F_1_ scores was merely 0.59% at the last epoch.

### ROC and PR curves for multi-class classification

**Figure 3:**
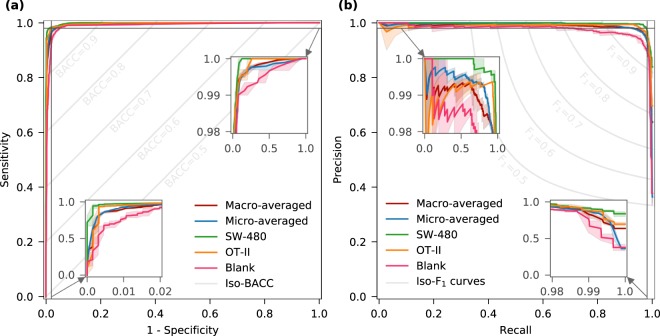
Receiver operating characteristics (ROC) and precision-recall (PR) curves for multi-class classification. (**a**) ROC curves, as evaluators of classifier output quality, are generated based on the predicted probabilities for the test dataset. They are shown for each class along with their macro-averaged and micro-averaged forms. ROC curves show the trade-off between classifier sensitivity and specificity, and an ideal ROC curve reaching the top left corner indicates both high sensitivity and specificity. Gray lines in the figure are the iso-BACC contour lines showing the BACC values from 0.5 to 0.9. (**b**) PR curve is another evaluator of classifier output quality, especially when dealing with the imbalanced classes. Precision is a measure of correctness, while recall, which is same as sensitivity, measures completeness. The PR curves for individual classes and their averaged forms are preferred to reach upper right corner, where both precision and recall are high. The iso-F_1_ contour lines show the F_1_ scores from 0.5 to 0.9. The shaded areas demonstrate the range of variations in each performance curve for five different training runs. The high sensitivity (recall), specificity, and precision regions of the ROC and PR curves are magnified in the insets for superior clarity.

To analyze classifier output quality, receiver operating characteristic (ROC) and precision-recall (PR) curves were utilized. ROC curves are typically employed to highlight the trade-off between sensitivity and specificity at different classification thresholds for a binary classifier. To extend the ROC curve to a multi-class classifier, ROC curves are drawn for each individual category and their macro-averaged and micro-averaged forms, and the robustness of these classifiers are quantitatively revealed by the area under the ROC curve (AUC). Accurate classifiers display regions with both high sensitivity and specificity in corresponding ROC curves with the AUC approximating 1.0 (i.e. 100%). To evaluate the accuracy of the model trained in this manuscript fairly, the model was used to process the test dataset and generate ROC curves (Fig. 3(a)). Data related to both the classes and the averaged forms demonstrates high quality classification, surpassing sensitivity/specificity values of 99.66%/99.37%. Based on AUC, the classification of *SW-480* (AUC = 99.75%) and *OT-II* (AUC = 99.50%) categories are slightly more robust than that of blank (AUC = 98.60%) category. The AUC is 99.36% for micro-averaged and is 99.34% for macro-averaged forms, both of which are satisfactory. To visualize balanced accuracy (BACC), which is the arithmetic mean of sensitivity and specificity, the iso-BACC contour lines from BACC = 0.5 to 0.9 are also shown in the ROC figure. It can be observed that all of these classifiers exceed 95% balanced accuracy. To demonstrate the trade-off between precision and recall, PR curves for the three individual categories and their averaged forms were generated (Fig. 3(b)). The PR curves for all these classifiers show precision/recall of above 97.36%/99.66%, and the robustness of the classifiers are described by the area under PR curve (AUCPR). The AUCPR is 98.76% for macro-averaged, 98.22% for micro-averaged, 99.57% for *SW-480*, and 98.87% for *OT-II* classifiers, while for blank classifier, the AUCPR is relatively small (96.22%), demonstrating the robustness of the model. Since the F_1_ score is the harmonic mean of precision and recall, the iso-F_1_ contour lines from F_1_ score = 0.5 to 0.9 are drawn, revealing that the F_1_ scores are greater than 93% for all of the classifiers.

### Learning curve

**Figure 4:**
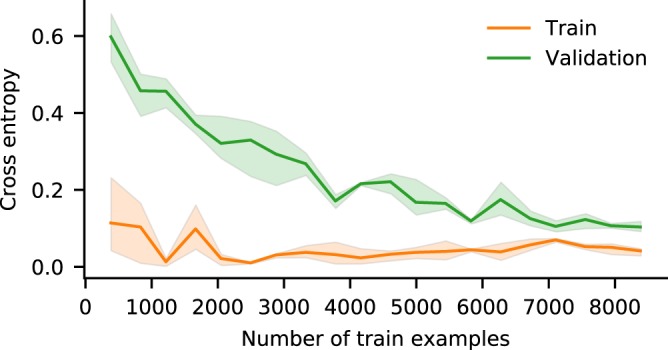
Learning curves. The performance of the model is evaluated at different numbers of train examples. We trained the neural network using part of the train dataset and observed the cross-entropy errors after 100 epochs of training. To calculate the validation cross-entropy errors, we used all of the examples in the validation dataset. If the train dataset size is very small, the model does not generalize well, and the validation cross-entropy error becomes very high. When more examples from the train dataset are used, the model can generalize much better, and the validation error decreases until it settles. The train and validation cross-entropy errors almost plateau beyond a certain number of train examples, which is around 6700 examples in this case.

In another experiment, the effect of varying the train dataset size is examined, i.e. learning curve (Fig. 4). The train cross-entropy error is measured after 100 epochs of training using part of train dataset, and the validation cross-entropy error is calculated by using all of the examples in the validation dataset. As the number of train examples increases, the validation cross-entropy error reduces and the model generalizes better. Since the train and validation learning curves converge at about 6700 train examples, our dataset has more than sufficient examples to train the proposed neural network model.

### Regularization

**Figure 5:**
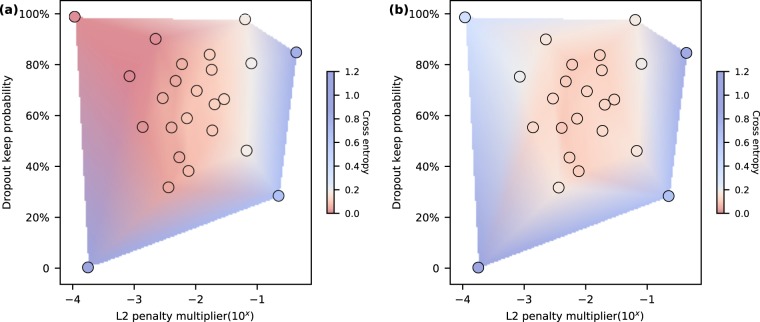
Regularization by L2 and dropout. Regularization is critical in balancing the trade-off between underfitting (bias) and overfitting (variance). The regularization techniques used in this model are L2 norm combined with dropout, which involve hyperparameters of L2 penalty multiplier and dropout keep probability (1 - dropout rate). By using random search, these two hyperparameters are explored, and the optimal point is used in the final training. The performance of regularization is evaluated by the last epoch validation cross entropy of the model with different pairs of regularization hyperparameters (each dot represents one pair of regularization pair). (**a**) The train cross entropy increases as either L2 multiplier or dropout rate is increased. (**b**) The validation cross entropy on the other hand is large at small L2 multiplier and dropout rate due to overfitting. The optimized regularization pair is determined by the minimal validation cross entropy.

Our model is regularized by the L2 and dropout techniques simultaneously. The L2 regularization method is a common regularizer adding a penalty equal to the sum of the squared magnitude of all parameters multiplied by a hyperparameter called the L2 penalty multiplier. Dropout is another form of regularization, which is applied following the fully-connected layers 1 and 2 of our neural network. Note that the dropout is only active in training iterations. The outputs of these two fully-connected layers are masked randomly with a keep probability hyperparameter, so that only part of the information is delivered to the next layer. Since L2 and dropout regularization techniques are blended in our training, random search is used to optimize both hyperparameters. Random search has been demonstrated to be more effective than grid search in hyperparameter optimization^58^. The search is staged from coarse to fine. At the coarse stage, twelve trials are carried out. The L2 penalty multiplier is randomly sampled from a uniform distribution between 10^−4^ and 10^0^, while dropout keep probability is chosen randomly from a uniform distribution between 0 and 100%. The cross-entropy errors of train and validation are observed to evaluate the performance of each regularizer and the results are shown by a pseudocolor plot of unstructured triangular grids (Fig. 5). Then the search is further narrowed to the region where optimal results are obtained and another twelve sets of hyperparameters are sampled. From the pseudocolor plot displaying all resultant trials, the optimized regularization hyperparameters within the search region locates at L2 penalty multiplier of 0.00408 and dropout keep probability of 55.14%, where the validation cross entropy is the minimal. The detailed hyperparameter settings of all trials are shown in Table 1.

**Table 1:** Hyperparameters for regularization.

Trial number (L2 penalty multiplier, dropout keep probability)		
1 (0.43498, 84.61%)	2 (0.01698, 83.74%)	3 (0.00085, 75.27%)
4 (0.22378, 28.34%)	5 (0.00771, 38.11%)	6 (0.06408, 97.57%)
7 (0.02045, 64.27%)	8 (0.00294, 66.68%)	9 (0.02925, 66.28%)
10 (0.08087, 80.31%)	11 (0.00011, 98.59%)	12 (0.00018, 0.23%)
13 (0.01049, 69.53%)	14 (0.00726, 58.78%)	15 (0.00225, 89.91%)
16 (0.00364, 31.68%)	17 (0.00139, 55.35%)	18 (0.06780, 46.09%)
19 (0.00546, 43.48%)	20 (0.01878, 53.98%)	21 (0.00603, 80.00%)
22 (0.01834, 77.79%)	23 (0.00476, 73.39%)	24 (0.00408, 55.14%)

## Discussion

**Figure 6:**
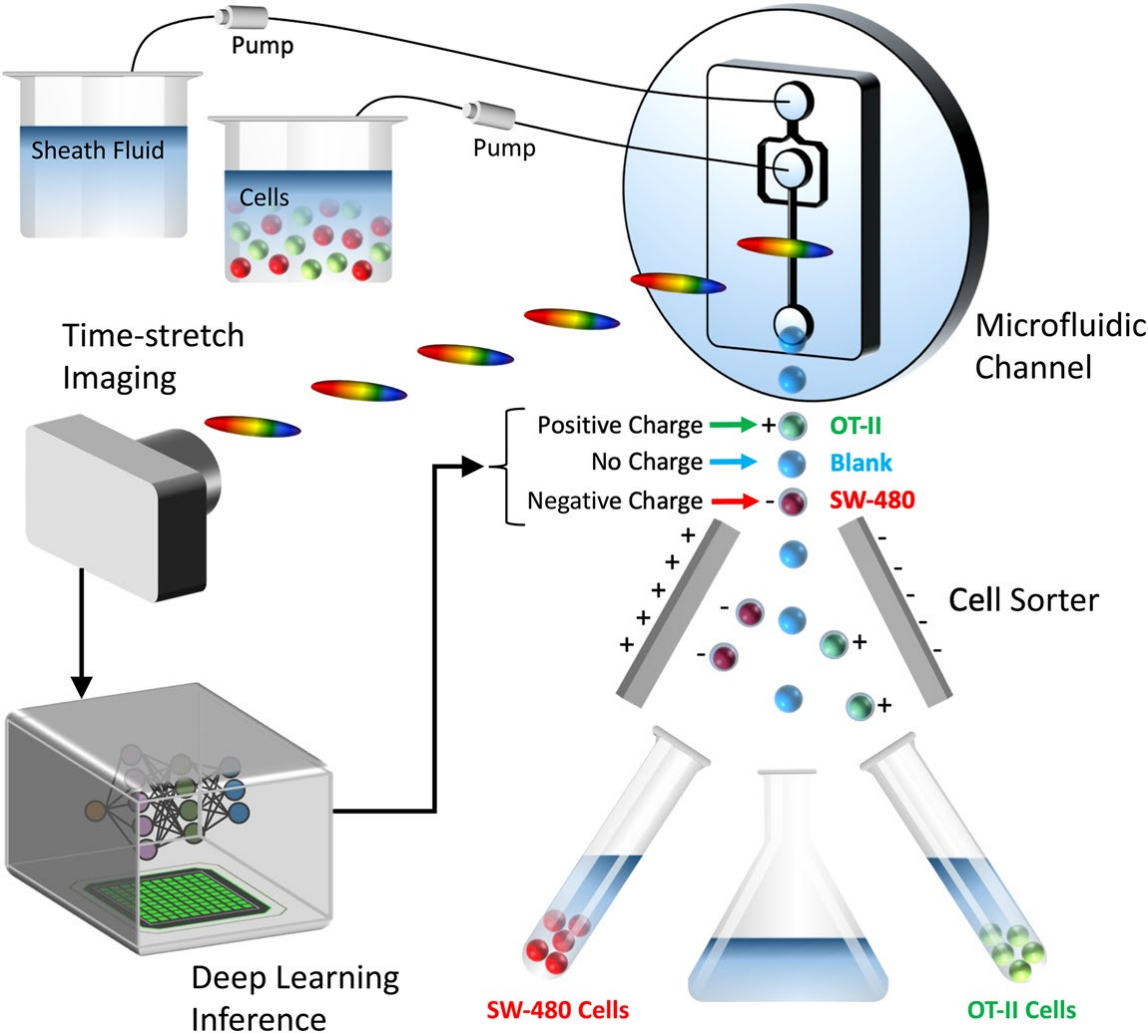
Deep cytometry: application of deep learning in cell sorting and flow cytometry. A microfluidic channel with hydrodynamic focusing mechanism uses sheath fluid to align the cells in the center of field-of-view. The rainbow pulses formed by the time-stretch imaging system capture line images of the cells in the channel, containing blur-free quantitative label-free images of the cells flowing at a high speed. The output waveforms of the time-stretch imaging system are directly passed to a deep neural network without any signal processing. The network achieves rapid cell classification with high accuracy, fast enough to make decisions before the cells reach the sorting mechanism. Different types of cells are categorized and charged with different polarity charges so that they can be separated into different collection tubes.

In order for label-free real-time imaging flow cytometry to become a feasible methodology, imaging, signal processing, and data analysis need to be completed while the cell is traveling the distance between the imaging point (field-of-view of the camera) in the microfluidic channel and the cell sorting mechanism (Fig. 6). During imaging, the time-stretch imaging system is used to rapidly capture the spatial information of cells at high throughput. A train of rainbow flashes illuminates the target cells as line scans. The features of the cells are encoded into the spectrum of these optical pulses, representing one-dimensional frames. Pulses are stretched in a dispersive optical fiber, mapping their spectrum to time. They are sequentially captured by a photodetector, and converted to a digital waveform, which can be analyzed by the neural network. The imaging and data capture take less than 0.1 ms for each waveform element, which covers a field-of-view of 25 *μm* in the channel direction, often containing only one cell surrounded by the suspension buffer or no cell. So, the delay in making a decision for cell sorting is dominated by the data processing time of the neural network.

To quickly classify the target cells based on the collected data, we demonstrate the utility of analyzing waveforms directly by a deep neural network, referred to as deep cytometry. The classification model is trained offline using datasets for the target cell types, and then used in an online system for cell sorting. The processing time of this model (the latency for inference of a single-example batch by a previously trained model) is 23.2 ms per example using an Intel Xeon CPU (8 cores), 8.6 ms per example on an NVIDIA Tesla K80 GPU, and 3.6 ms per example on an NVIDIA Tesla P100 GPU (Table 2). Thus, for our setup with the cell flow rate of 1.3 m/s in the microfluidic channel, the cells travel 30.2 mm for the Intel CPU, 11.2 mm for the NVIDIA K80 GPU, or 4.7 mm for the NVIDIA P100 GPU before the classification decision is made. So, the microfluidic channels should be at least as long as these cell travel distances. Fabrication of microfluidic channels beyond these length limits is very practical, and the cells can remain ordered within such short distances. Therefore, the type of each cell can be determined by our model in real-time before it reaches the cell sorter. Oftentimes the flow speed is less than our setup, and the length limitation is further relaxed.

**Table 2:** Inference processing time on different hardware (ms/example).

Batch size (no. of examples)	Intel Xeon CPU	NVIDIA K80 GPU	NVIDIA P100 GPU
1	23.2	8.6	3.6
2	21.0	5.7	2.0
4	12.9	4.3	1.6
8	10.0	3.7	1.3
16	8.7	2.9	0.8
32	8.0	2.6	0.7
64	7.4	1.9	0.6

Besides the time-stretch imaging signals used in the demonstrations here, our deep learning approach for real-time analysis of flow cytometry waveforms, namely deep cytometry, can also be applied to the signals captured by other sensors such as CMOS (complementary metal-oxide semiconductor) or CCD (charge-coupled device) imagers, photomultiplier tubes (PMTs), and photodiodes.

## Conclusion

In this manuscript, a deep convolutional neural network with fast inference for direct processing of flow cytometry waveforms was presented. The results demonstrate record performance in label-free detection of cancerous cells with a test F_1_ score of 95.71% and accuracy of 95.74% with high consistency and robustness. The system achieves this accurate classification in less than a few milliseconds, opening a new path for real-time label-free cell sorting.

## Methods

### Microfluidic channel

To fulfill the requirement of next generation cell sorting, microfluidic chip devices have become a promising solution due to their capability of precise flow manipulation and control^25^. We have designed and fabricated a unique microfluidic channel with a dielectric-mirror substrate to quantitatively image the cells in our setup. The cell samples were injected from the inlet and then hydrodynamically focused under the sheath fluid pressure at the center of the channel, lining up in the camera field-of-view. The channel height is high enough to allow the passage of the cells without frequent cloggage, but sufficiently low to keep the cells in depth of focus, while they are being imaged by the TS-QPI system. By carefully choosing the injection rates of sheath and sample fluids, the cell flow rate was controlled at 1.3 m/s to realize high throughput cell analysis.

### Deep learning algorithm for cell classification

To classify the cell types and determine the polarity of the charges added to the cells in the conventional sorting mechanisms, a deep learning algorithm is used. The deep convolutional neural network is trained end-to-end with the collected time-series data carrying the information of *SW-480* cells, *OT-II* cells, and blank waveform elements with no cells. Between the layers, the nonlinearity is introduced by the rectified linear unit (ReLU) function *f*(*x*) = *max*(0, *x*), which is typically used in ConvNets. After the logits are obtained, we use softmax function to achieve predicted probabilities of each class

1 $${p}_{i,c}=\frac{{e}^{{l}_{i,c}}}{{\sum }_{c^{\prime} =1}^{3}\,{e}^{{l}_{i,c\text{'}}}},\,c=1,\cdots ,3$$

here *l*_*i*,*c*_ is the logit of each class *c*, for example *i*. The cross-entropy loss for multi-class is calculated in the forward propagation as

2 $${L}_{{\rm{c}}{\rm{r}}{\rm{o}}{\rm{s}}{\rm{s}}{\textstyle  \mbox{-} }{\rm{e}}{\rm{n}}{\rm{t}}{\rm{r}}{\rm{o}}{\rm{p}}{\rm{y}}}=-\frac{1}{N}\sum _{i=0}^{N-1}\,\sum _{c=1}^{3}\,{y}_{i,c}\,{\rm{l}}{\rm{o}}{\rm{g}}\,({p}_{i,c})$$

in which *y*_*i*,*c*_ is the one-hot (1-of-3) binary indicator presenting the true label of example *i*, and *N* is the number of dataset examples. We first searched a good learning rate for Adam optimizer^56^ based on the train and validation cross-entropy convergence. Then the hyperparameters for the regularization were finely tuned by random search. The model was fully trained at each searching point, and the best model with optimized hyperparameters was selected based on the minimum validation cross entropy. At the cell classification stage, the pretrained model was employed to categorize the cell samples with forward propagation, which obtains a very short inference time. Thus, real-time decision can be made before the cell samples pass to the cell sorter.

### Label-free cell sorting mechanism

Since real-time cell classification with high accuracy is achieved by our neural network, the flow cytometer system can be upgraded to perform cell sorting. The target cells can be further analyzed by downstream methods such as DNA sequencing, after the purification and collection by the cell sorter. A common way to capture the target cells is applying different polarities of charges to the drops that contain different types of cells according to the decision made by the cell classification system^59^. For example, the drops containing *SW-480* cells are charged with negative charges, while the *OT-II* cell drops are charged with positive charges and the blank drops with no cells inside get no charge. When those drops are passing through the two sorter plates which are charged with positive and negative charges, the drops are separated into two collection tubes by the electrical force because of their different charge polarities and the blank drops go to the waste collection bucket (Fig. 6).

### Data analytic tools

The deep convolutional neural network was implemented by Python 3.5.3 API of TensorFlow 1.14.0^60^. The performance of the convolutional model was analyzed on three types of virtual machines on Google Cloud Platform. One machine used 8 Intel Xeon CPU cores clocking at 2.2 GHz, 52 GB of memory, and Intel MKL-DNN libraries. The other two machines were also supplied with a single NVIDIA Tesla K80 GPU and a single NVIDIA Tesla P100 GPU configured with CUDA Toolkit 10.0 and cuDNN v7.4.1. The NVIDIA Tesla K80 GPU accelerates the forward propagation compared with the Intel CPU. However, NVIDIA Tesla P100 GPU can reduce the inference time even more, due to its unique high-performance computing Pascal architecture. The inference times for different machines when evaluated on the test dataset are shown in Table 2.

### Time-stretch imaging

Unlike CMOS (complementary metal-oxide semiconductor) or CCD (charge-coupled device) chips commonly used in other imaging flow cytometers, our system utilizes a time-stretch imaging device. A mode-locked laser generates optical pulses at a repetition rate of 36.6 MHz with about 100 fs pulse width. The spectrum of the pulses is centered at 1565 nm wavelength with a bandwidth of about 30 nm, but the power spectral density of the pulses is very nonuniform across the bandwidth and not suitable for our imaging system. To resolve this, the bandwidth of the pulses is broadened by a highly nonlinear fiber (nonlinear coefficient of 11.5 W^−1^ km^−1^, attenuation of 0.90 dB/km) to about 100 nm (1505 nm to 1605 nm), and only the flat spectrum from 1581 nm to 1601 nm is passed by a wavelength division multiplexer (WDM) filter to the time-stretch imaging system. Also, to amplify the pulses using an erbium doped fiber amplifier (EDFA) with minimal spectral distortion, they are linearly chirped by a short dispersion compensating fiber (DCF with about 60 ps/nm dispersion). The pulses are directed by an optical circulator to the diffraction gratings, causing the pulses to be spatially dispersed like rainbow flashes. The rainbow pulses are split into two paths (arms) by the beam splitter of a Michelson interferometer. In one path, the pulses illuminate the target cells, and the spatial information of the cells are encoded into the pulses. The rainbow pulses and their original forms are reflected by the dielectric mirrors at the end of the Michelson interferometer arms and interfere in the beam splitter. Their interference patterns go back to the circulator and are guided toward a dispersive fiber. The interfered pulses are stretched in time by an amplified time-stretch dispersive Fourier transform system, which consists of a dispersion compensating fiber, Raman pump lasers, and wavelength division multiplexers. The amplified time-stretch pulses are detected by a 10 Gb/s photodetector (Discovery Semiconductors DSC-402APD) and converted to digital time-series data by an analog-to-digital converter (Tektronix DPO72004C) with 50 GS/s sampling rate and 20 GHz bandwidth.

### Metrics

To evaluate the classification performance in different forms, we calculated several metrics for comparison. Among these metrics, F_1_ score can be calculated as the harmonic mean of the precision and the recall

3 $${F}_{1}=2\times \frac{{\rm{precision}}\times {\rm{recall}}}{{\rm{precision}}+{\rm{recall}}}$$

where

4 $${\rm{p}}{\rm{r}}{\rm{e}}{\rm{c}}{\rm{i}}{\rm{s}}{\rm{i}}{\rm{o}}{\rm{n}}=\frac{{\rm{t}}{\rm{r}}{\rm{u}}{\rm{e}}\,{\rm{p}}{\rm{o}}{\rm{s}}{\rm{i}}{\rm{t}}{\rm{i}}{\rm{v}}{\rm{e}}}{{\rm{t}}{\rm{r}}{\rm{u}}{\rm{e}}\,{\rm{p}}{\rm{o}}{\rm{s}}{\rm{i}}{\rm{t}}{\rm{i}}{\rm{v}}{\rm{e}}+{\rm{f}}{\rm{a}}{\rm{l}}{\rm{s}}{\rm{e}}\,{\rm{p}}{\rm{o}}{\rm{s}}{\rm{i}}{\rm{t}}{\rm{i}}{\rm{v}}{\rm{e}}}$$

5 $${\rm{r}}{\rm{e}}{\rm{c}}{\rm{a}}{\rm{l}}{\rm{l}}=\frac{{\rm{t}}{\rm{r}}{\rm{u}}{\rm{e}}\,{\rm{p}}{\rm{o}}{\rm{s}}{\rm{i}}{\rm{t}}{\rm{i}}{\rm{v}}{\rm{e}}}{{\rm{t}}{\rm{r}}{\rm{u}}{\rm{e}}\,{\rm{p}}{\rm{o}}{\rm{s}}{\rm{i}}{\rm{t}}{\rm{i}}{\rm{v}}{\rm{e}}+{\rm{f}}{\rm{a}}{\rm{l}}{\rm{s}}{\rm{e}}\,{\rm{n}}{\rm{e}}{\rm{g}}{\rm{a}}{\rm{t}}{\rm{i}}{\rm{v}}{\rm{e}}}$$

Since we are dealing with a multi-class problem, we need to consider the averaged F_1_ score of the classes. For micro-averaged F_1_ score, the total number of true positive, false positive, and false negative are calculated globally to obtain the ultimate precision and recall:

6 $${\rm{micro}} \mbox{-} {\rm{averaged}}\,{\rm{precision}}=\frac{{\sum }_{c=1}^{3}\,{\rm{true}}\,{{\rm{positive}}}_{c}}{{\sum }_{c=1}^{3}\,{\rm{true}}\,{{\rm{positive}}}_{c}+{\sum }_{c=1}^{3}\,{\rm{false}}\,{{\rm{positive}}}_{c}}$$

7 $${\rm{micro}} \mbox{-} {\rm{averaged}}\,{\rm{recall}}=\frac{{\sum }_{c=1}^{3}\,{\rm{true}}\,{{\rm{positive}}}_{c}}{{\sum }_{c=1}^{3}\,{\rm{true}}\,{{\rm{positive}}}_{c}+{\sum }_{c=1}^{3}\,{\rm{false}}\,{{\rm{negative}}}_{c}}$$

8 $${\rm{m}}{\rm{i}}{\rm{c}}{\rm{r}}{\rm{o}}{\textstyle  \mbox{-} }{\rm{a}}{\rm{v}}{\rm{e}}{\rm{r}}{\rm{a}}{\rm{g}}{\rm{e}}{\rm{d}}\,{F}_{1}=2\times \frac{{\rm{m}}{\rm{i}}{\rm{c}}{\rm{r}}{\rm{o}}{\textstyle  \mbox{-} }{\rm{a}}{\rm{v}}{\rm{e}}{\rm{r}}{\rm{a}}{\rm{g}}{\rm{e}}{\rm{d}}\,{\rm{p}}{\rm{r}}{\rm{e}}{\rm{c}}{\rm{i}}{\rm{s}}{\rm{i}}{\rm{o}}{\rm{n}}\times {\rm{m}}{\rm{i}}{\rm{c}}{\rm{r}}{\rm{o}}{\textstyle  \mbox{-} }{\rm{a}}{\rm{v}}{\rm{e}}{\rm{r}}{\rm{a}}{\rm{g}}{\rm{e}}{\rm{d}}\,{\rm{r}}{\rm{e}}{\rm{c}}{\rm{a}}{\rm{l}}{\rm{l}}}{{\rm{m}}{\rm{i}}{\rm{c}}{\rm{r}}{\rm{o}}{\textstyle  \mbox{-} }{\rm{a}}{\rm{v}}{\rm{e}}{\rm{r}}{\rm{a}}{\rm{g}}{\rm{e}}{\rm{d}}\,{\rm{p}}{\rm{r}}{\rm{e}}{\rm{c}}{\rm{i}}{\rm{s}}{\rm{i}}{\rm{o}}{\rm{n}}+{\rm{m}}{\rm{i}}{\rm{c}}{\rm{r}}{\rm{o}}{\textstyle  \mbox{-} }{\rm{a}}{\rm{v}}{\rm{e}}{\rm{r}}{\rm{a}}{\rm{g}}{\rm{e}}{\rm{d}}\,{\rm{r}}{\rm{e}}{\rm{c}}{\rm{a}}{\rm{l}}{\rm{l}}}$$

Alternatively, macro-averaged F_1_ score calculates the metrics for each class and assigns the same weights to them,

9 $${{\rm{precision}}}_{c}=\frac{{\rm{true}}\,{{\rm{positive}}}_{c}}{{\rm{true}}\,{{\rm{positive}}}_{c}+{\rm{false}}\,{{\rm{positive}}}_{c}}$$

10 $${{\rm{recall}}}_{c}=\frac{{\rm{true}}\,{{\rm{positive}}}_{c}}{{\rm{true}}\,{{\rm{positive}}}_{c}+{\rm{false}}\,{{\rm{negative}}}_{c}}$$

11 $${\rm{macro}} \mbox{-} {\rm{averaged}}\,{\rm{precision}}=\frac{1}{3}\sum _{c=1}^{3}\,{{\rm{precision}}}_{c}$$

12 $${\rm{macro}} \mbox{-} {\rm{averaged}}\,{\rm{recall}}=\frac{1}{3}\sum _{c=1}^{3}\,{{\rm{recall}}}_{c}$$

13 $${\rm{macro}} \mbox{-} {\rm{averaged}}\,{F}_{1}=\frac{1}{3}\sum _{c=1}^{3}\,2\times \frac{{{\rm{precision}}}_{c}\times {{\rm{recall}}}_{c}}{{{\rm{precision}}}_{c}+{{\rm{recall}}}_{c}}$$

while weighted-averaged F_1_ score assigns different weights, *w*_*c*_, which are determined by the number of the examples for each true label^61^,

14 $${\rm{condition}}\,{{\rm{positive}}}_{c}={\rm{true}}\,{{\rm{positive}}}_{c}+{\rm{false}}\,{{\rm{negative}}}_{c}$$

15 $${\rm{condition}}\,{{\rm{negative}}}_{c}={\rm{true}}\,{{\rm{negative}}}_{c}+{\rm{false}}\,{{\rm{positive}}}_{c}$$

16 $${w}_{c}=\frac{{\rm{condition}}\,{{\rm{positive}}}_{c}}{{\rm{condition}}\,{{\rm{positive}}}_{c}+{\rm{condition}}\,{{\rm{negative}}}_{c}}$$

17 $${\rm{weighted}} \mbox{-} {\rm{averaged}}\,{\rm{precision}}=\sum _{c=1}^{3}\,{w}_{c}\times {{\rm{precision}}}_{c}$$

18 $${\rm{weighted}} \mbox{-} {\rm{averaged}}\,{\rm{recall}}=\sum _{c=1}^{3}\,{w}_{c}\times {{\rm{recall}}}_{c}$$

19 $${\rm{weighted}} \mbox{-} {\rm{averaged}}\,{F}_{1}=\sum _{c=1}^{3}\,2{w}_{c}\times \frac{{{\rm{precision}}}_{c}\times {{\rm{recall}}}_{c}}{{{\rm{precision}}}_{c}+{{\rm{recall}}}_{c}}$$

Accuracy is a traditional metric that gives the fraction of correct predictions,

20 $${\rm{accuracy}}(y,y^{\prime} )=\frac{1}{N}\sum _{i=0}^{N-1}\,1(y{\text{'}}_{i}={y}_{i})$$

where *y*′_*i*_ represents the predicted value of the i-th sample, *y*_*i*_ is the corresponding true label, and 1(...) is the indicator function. Due to the imbalance which may exist in the data, we also consider the balanced accuracy (BACC), which is same as averaged recall. The averaged recall can be calculated in different forms as seen in Eqs 7, 12, and 18, where the micro-averaged form is same as accuracy. Finally, cross-entropy, which has been previously explained in Eq. 2, is a differentiable metric for monitoring the classifier.

## Supplementary information

Supplementary Information

## Data Availability

The authors confirm that the data supporting the findings of this study are available within the article and its Supplementary Materials.
