# Supplementary material for: Deep Cytometry: Deep learning with Real-time Inference in Cell Sorting and Flow Cytometry
Source: Sci Rep. 2019 Jul 31;9:11088. doi: 10.1038/s41598-019-47193-6 (PMC6668572; doi:10.1038/s41598-019-47193-6)
Supplement: Supplementary file 1 — Supplementary Information [file 41598_2019_47193_MOESM1_ESM.pdf]

Supplementary Information

Deep Cytometry:
Deep Learning with Real-time Inference in Cell Sorting and Flow Cytometry

Yueqin Li, Ata Mahjoubfar, Claire Lifan Chen, Kayvan Reza Niazi, Li Pei, and Bahram Jalali

Supplementary Note 1: Confusion matrix To further evaluate the output quality of the classifier, a confusion matrix on the test dataset is used for visualization of the predictions (Fig. 1). At the first several epochs, the quality of the classifier is not very good because the model has not learned very well. As the number of epochs increases, the neural network is more finely trained, thus the confusion matrix has higher diagonal values and less off-diagonal elements, indicating more correct predictions. The classifier can predict very well after the training reaching epoch 60, and at the last epoch the three classes are almost perfectly categorized. Some *SW480* and *OT-II* cells are mislabeled as blanks and a few blank examples are misclassified as cells. These confusions may be caused by the ambiguous threshold of labeling examples as blanks during data preparation.

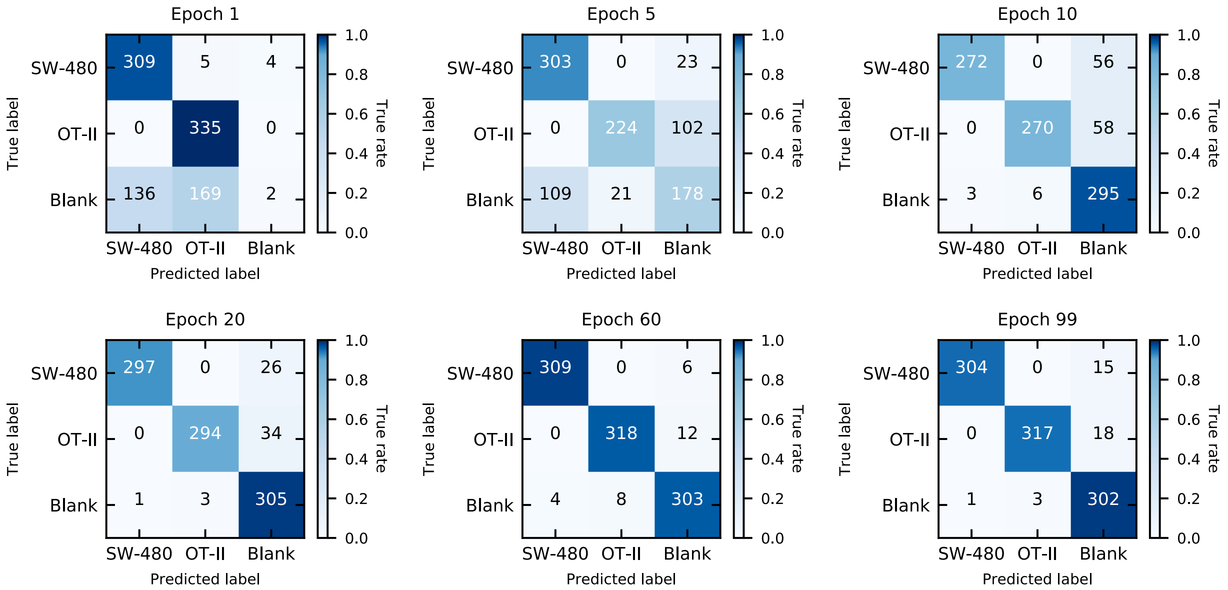

Supplementary Figure 1: **Confusion Matrix.** Confusion matrix is an evaluator of output quality of classifier, which is very intuitive for visualizing the classification performance. The number of diagonal elements represents the equality of predicted labels and true labels, while the off-diagonal elements are the number of examples which are wrongly predicted by the classifier. At the end of every epoch, the trained model is evaluated on the test dataset, and the results of epoch 1, 5, 10, 20, 60 and 99 are shown as examples.

Supplementary Note 2: Balanced accuracy of the training Except for F_1 score, we also use balanced accuracy to measure the performance of the model by monitoring every training epoch. It demonstrates a similar trend as the F_1 score measure. The neural network works well in categorizing the cells (*SW-480* and *OT-II*) and the overall performance is good when measured in balanced accuracy. The validation balanced accuracy rises gradually and settles beyond epoch 60,

and the weighted-averaged balanced accuracy for the validation can achieve 97.00%.

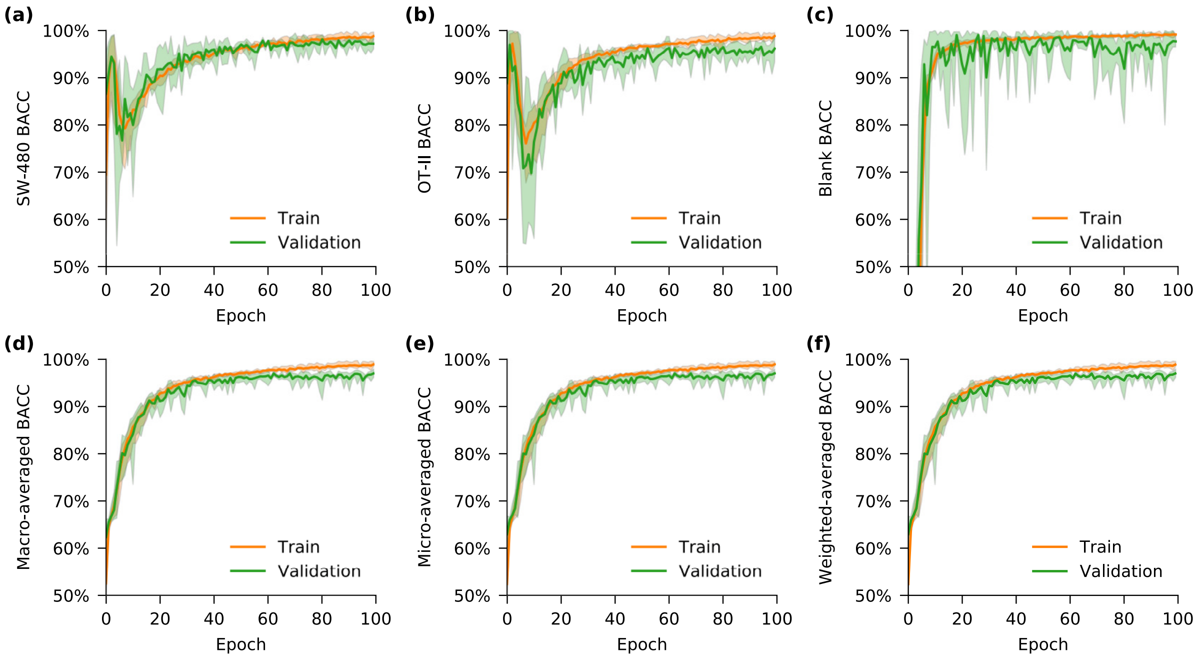

Supplementary Figure 2: **Balanced accuracy of the training.** Balanced accuracy is another way to evaluate the learning performance of the neural network. The balanced accuracy for the three individual categories and their averaged forms are calculated over all the training epochs. Similar to the performance measured by F_1 score, our neural network can well classify (a) *SW-480* and (b) *OT-II* cells while have some difficulties in identifying (c) Blanks at the beginning. The overall balanced accuracies improve and then become steady beyond a point.

Supplementary Note 3: Final epoch measures for all metrics There are multiple metrics that can be used to evaluate the classification performance. To have a more comprehensive sense of the output quality of the classifier, we summarize the final epoch results for a set of these metrics

Supplementary Table 1: **Final epoch measures for all metrics**

Metrics type	Train		Validation		Test	
	mean	S.D.	mean	S.D.	mean	S.D.
F_1-macro	98.88%	0.34%	96.99%	0.59%	95.71%	1.08%
F_1-micro	98.87%	0.34%	97.00%	0.60%	95.71%	1.08%
F_1-weighted	98.87%	0.34%	97.01%	0.59%	95.71%	1.08%
Accuracy	98.87%	0.34%	97.00%	0.60%	95.74%	1.10%
BACC-macro	98.87%	0.34%	96.99%	0.60%	95.74%	1.10%
BACC-micro	98.87%	0.34%	97.00%	0.60%	95.74%	1.10%
BACC-weighted	98.87%	0.34%	97.00%	0.60%	95.74%	1.10%
Cross entropy	0.0359	0.0101	0.1150	0.0209	0.1631	0.0389

(including F_1 score, accuracy, balanced accuracy and cross entropy), and list their mean values and standard deviations (S.D.) in Table 1 for 5 runs initialized with different random starting weights and biases. The overall performance of the deep neural network evaluated by all of these measures is satisfactory, providing a promising prospect for direct analysis of the sensor waveforms.
